# Aberrant Brain Entropy in Alzheimer's Disease: Evidence From a Resting‐State fMRI Study

**DOI:** 10.1002/brb3.71454

**Published:** 2026-04-30

**Authors:** Xuke Zhang, Meihai Wen, Simin Zhang, Chen Rao, Tong Gu, Zhiwen Zha, Chen He, Yuanyuan Song, Shuang Niu, Lei Zhu, Chuanqing Yu

**Affiliations:** ^1^ Bengbu Medical University Bengbu Anhui Province China; ^2^ Department of Neurology The First Affiliated Hospital of Anhui University of Science and Technology (The First People's Hospital of Huainan) Huainan Anhui Province China

**Keywords:** Alzheimer's disease (AD), brain entropy (BEN), cognitive impairment, resting‐state functional magnetic resonance imaging (rs‐fMRI)

## Abstract

**Background:**

The neurodegenerative disorder, Alzheimer's disease (AD), is typified by insidious onset and increasing prevalence with advancing age. It primarily causes comprehensive functional decline in memory, language, and executive control. The scarcity of reliable imaging biomarkers has led to resting‐state functional magnetic resonance imaging (rs‐fMRI) becoming a seminal tool for investigating functional alterations in neurological disorders. Brain entropy (BEN) is an indicator derived from rs‐fMRI data, quantifying the complexity of functional brain activity and the uncertainty of spontaneous activity. This study aims to utilize BEN technology to elucidate the spatial distribution characteristics of functional complexity within the brains of AD patients.

**Methods:**

The present study comprised 30 patients diagnosed with AD and 46 healthy controls recruited from the same center. A comprehensive neuropsychological assessment and rs‐fMRI data acquisition were carried out for all participants. Intergroup comparisons were conducted using voxel‐level BEN value maps. To this end, a partial correlation analysis was conducted with the objective of ascertaining the association between BEN values in regions exhibiting significant intergroup differences and cognitive function scores.

**Result:**

This study found significantly decreased BEN in the right superior temporal gyrus, right inferior frontal gyrus, and left posterior parietal gyrus among patients with AD, which was significantly correlated with MMSE and MoCA cognitive scores. Exploratory analyses also revealed abnormal trends in the precuneus, cuneus, and left frontotemporal pole.

**Conclusion:**

A widespread reduction in BEN was observed in cerebral regions implicated in language memory, processing, and integrative functions, consistent with cognitive decline in AD. As a sensitive and noninvasive metric, BEN shows promise as an imaging marker for early screening and monitoring of AD.

## 1 Introduction

It is estimated that between 60% and 70% of all cases of dementia are attributable to Alzheimer's disease (AD), with the condition representing the foremost cause of dementia (2024 Alzheimer's Disease Facts and Figures). With the acceleration of global population ageing, an estimated 55 million individuals are currently affected by AD worldwide, Current projections indicate a predicted increase in the number of individuals to be 78 million by 2030 and 139 million by 2050 (Shin 2022). As an incurable neurodegenerative disorder, AD markedly elevates the risk of disability, increases disease burden, and imposes substantial strain on healthcare systems. Previous studies have demonstrated that certain pharmacological agents, including acetylcholinesterase inhibitors and NMDAR antagonists, may modestly delay disease progression; however, an effective cure remains unavailable (Chin et al. 2022; Xing et al. 2025). The pathophysiology of AD is multifactorial, including Aβ accumulation, tau protein hyperphosphorylation, synaptic impairment, and neuronal injury (Koychev et al. 2020). Nevertheless, significant challenges persist in early diagnosis, disease surveillance, and therapeutic evaluation across different stages of AD. Currently available neuroimaging modalities, such as PET, are constrained by limited spatial resolution and high costs, while cerebrospinal fluid analysis is invasive, restricting large‐scale implementation; cognitive assessment tools such as MMSE are additionally affected by limited patient compliance and susceptibility to subjective influences. Consequently, the identification of more precise, noninvasive, and economically feasible approaches for detection and monitoring is urgently required to enable early diagnosis, timely treatment, and effective intervention in AD.

As a noninvasive approach for investigating brain function, resting‐state functional magnetic resonance imaging (rs‐fMRI) enables continuous acquisition of temporal fluctuations in BOLD signals across brain regions during rest, providing a valuable means to characterize the temporal properties of brain functional activity. Evidence from neurobiology and computational neuroscience indicates that normal brain function relies on a dynamic equilibrium between functional differentiation and functional integration of neuronal activity (Tononi and Edelman 1998): distinct brain regions exhibit relatively independent specialized roles, while coordinated activation across regions supports integrated network function, together constituting “brain complexity.” During neurodegenerative processes, synaptic degeneration, network reconfiguration, and disruption of long‐range connectivity perturb this equilibrium, leading neuronal activity patterns to become either excessively synchronized and rigid, yielding a highly predictable low‐complexity ordered state, or fragmented into noisy and inefficient low‐complexity disordered states; both patterns are reflected by an overall reduction in the temporal complexity of brain signals. Consistent evidence from electroencephalogram (EEG), magnetoencephalography (MEG), and fMRI studies (Mandal et al. 2018; Sun et al. 2020) has demonstrated that signal complexity in patients with AD and mild cognitive impairment is markedly reduced relative to that in cognitively healthy older adults, predominantly involving regions associated with higher order cognitive processing, including the frontal, temporal, and parieto‐occipital lobes.

Entropy is a classical metric in information theory used to characterize uncertainty and diversity, and when applied to neural signals, it converts time‐series complexity into comparable quantitative measures. The BEN approach derived from rs‐fMRI extends this concept to the voxel and regional levels by calculating metrics such as the sample entropy of BOLD time series, thereby capturing the temporal irregularity and functional reserve of localized neural activity patterns. Prior investigations have demonstrated a generalized reduction in BEN across the AD spectrum, with diminished complexity observed in the medial temporal lobe, default mode network (DMN), frontal lobe, and occipital lobe, and significant associations with cognitive performance, indicating that attenuation of functional complexity may represent a shared mechanism underlying functional decline in AD (Z. Wang and Alzheimer's Disease Neuroimaging Initiative 2020; B. Wang et al. 2017). Previous studies, such as those by Niu et al. (2018) and Grieder et al. (2018), employed the multiscale entropy (MSE) algorithm to investigate patients with AD and reported alterations in complexity across multiple brain regions. However, it is worth noting that MSE differs significantly from the single‐scale entropy (BEN) utilized in this study, which is based on the BENtbx toolkit, in terms of computational principles and parameters. As pointed out by Lu and Wang (2021), the MSE method may exhibit systematic biases. In contrast, BEN has been proven to offer a more stable and reliable measure of brain functional complexity (Z. Wang and Alzheimer's Disease Neuroimaging Initiative 2020; Song et al. 2019). Therefore, although both the present study and MSE research address “brain complexity,” due to methodological disparities, direct comparisons of their results should be avoided. The choice of BEN in this study aims to provide a more stable and unbiased perspective for observing changes in resting‐state brain functional activity in AD patients.

Brain entropy (BEN) is a metric used to characterize the irregularity/complexity of resting‐state BOLD signal time series, compared to traditional functional indicators that mainly reflect local amplitude (such as ALFF/fALFF) or perfusion levels (such as resting‐state CBF), BEN provides supplementary information that is related to, but not entirely the same as, these indicators (Song et al. 2019). Thereby offering a distinct advantage in characterizing the transition from diverse to increasingly uniform functional states during neurodegenerative processes (Cieri et al. 2021).

Nevertheless, several limitations remain in current BEN research in AD. First, most investigations have relied on whole‐brain mean values or coarse region‐of‐interest (ROI)–level BEN metrics, or have reported entropy alterations confined to a limited number of regions (Niu et al. 2018; Grieder et al. 2018), These studies use the MSE algorithm, whereas we use the single‐scale entropy (BENtbx) algorithm, which differ in calculation methods and parameters. Therefore, it is difficult to directly compare the results of the two. The single‐scale entropy in our study avoids these biases and has been demonstrated to be stable and reliable in multiple studies. Second, prior work has primarily examined associations between BEN and global cognitive performance or a single assessment scale, such as MMSE (Sun et al. 2020; Liu et al. 2013), without comprehensive evaluation of the regionally specific relationships between BEN and multiple cognitive domains, including executive function, disease severity, and activities of daily living. Consequently, the correspondence between regional complexity reduction and distinct cognitive impairments remains insufficiently defined. On this basis, voxel‐wise BEN was calculated from 3.0T rs‐fMRI data in patients with AD and age‐ and sex‐matched healthy controls (HCs) to address the following objectives: (1) to determine whether BEN reduction is widespread and systematic in AD; (2) to delineate the spatial distribution pattern of BEN decreases across the whole brain; and (3) to assess associations between BEN and multiple neuropsychological measures, including MMSE, MoCA, ADAS‐Cog, CDR, and ADL, within regions showing significant alterations, thereby establishing a mapping between functional complexity impairment and specific cognitive domain deficits in AD. Through this approach, a more robust theoretical framework and empirical support are intended to be provided for the application of BEN in early screening, disease staging, and treatment response evaluation in AD.

## 2 Materials and Methods

### 2.1 Participants

The research protocol was approved by the Ethics Committee of the First Hospital of Anhui University of Science and Technology. Thirty patients with AD and 46 age‐ and sex‐matched HCs were enrolled. Written informed consent was obtained from all participants. Patients in the AD group were clinically diagnosed by experienced neurologists based on routine clinical assessment and established clinical diagnostic procedures available at our center. Because of practical limitations, cerebrospinal fluid biomarker testing and amyloid/tau PET imaging were not available for this cohort. Therefore, the diagnosis in the present study should be interpreted as a clinically established AD diagnosis rather than a biomarker‐confirmed biological diagnosis under the 2024 revised criteria. Plasma Aβ testing was available in all of the participants and is reported as supportive biomarker information.

Inclusion criteria for AD patients with cognitive impairment:

1. Age > 45 years;
2. Fulfillment of clinical presentation criteria, including progressive cognitive decline predominantly characterized by memory impairment, with possible concomitant deficits in executive function, language, or visuospatial abilities, along with deterioration in activities of daily living relative to baseline;
3. Cognitive assessment indicating mild to moderate impairment, defined by an MMSE score of 18–26;
4. Exclusion of alternative etiologies of cognitive impairment, such as cerebrovascular disease, Parkinson's disease, or depression.

**Exclusion criteria**:

1. Presence of other forms of dementia, including vascular dementia or frontotemporal dementia;
2. Severe psychiatric disorders, including schizophrenia or major depression;
3. Serious systemic or intracranial diseases, such as traumatic brain injury or neoplasms;
4. Substance dependence, including drug or alcohol abuse.

**Inclusion criteria for HC**:

1. Absence of subjective complaints related to cognitive impairment or memory decline;
2. Neuropsychological performance within the normal range, CDR = 0;
3. No family history of AD or other dementias;
4. Provision of written informed consent and eligibility for fMRI examination.

**Exclusion criteria for HC**:

1. History of neurological disorders, including stroke or Parkinson's disease;
2. History of psychiatric disorders, such as depression or schizophrenia;
3. Severe systemic conditions, including cardiac, hepatic, or renal disease, or malignancies;
4. Current or prior use of medications known to influence central nervous system function;
5. Presence of contraindications to MRI, including claustrophobia or metallic implants.

### 2.2 Magnetic Resonance Data Acquisition

Magnetic resonance imaging (MRI) data were collected using a 3.0‐Tesla scanner (Discovery MR750 3.0T). Participants were positioned supine in the scanner and instructed to remain motionless, awake, and relaxed while breathing normally. The application of earplugs and foam padding was implemented with the objective of minimizing acoustic noise and head motion.

rs‐fMRI data were collected via a single‐shot echo‐planar imaging sequence for BOLD imaging with the subsequent parameters: repetition time = 2000 ms, echo time = 30 ms, field of view = 220 × 220 mm^2^, flip angle = 90°, in‐plane matrix = 64 × 64, slice thickness = 4 mm, spatial resolution = 3.4 × 3.4 × 3.4 mm^3^, and 49 axial slices acquired without interslice gap.

Structural images for each participant were acquired using a three‐dimensional T1‐weighted magnetization‐prepared rapid gradient echo sequence, in order to obtain high‐resolution anatomical images. The following parameters were used for the acquisition: FOV = 256 × 256 mm, slice thickness = 1 mm without gap, TR = 7.45 ms, TE = 2.75 ms, and voxel size of 1 × 1 × 1 mm.

### 2.3 Data Preprocessing

Image preprocessing was conducted using the rs‐fMRI data processing assistant in combination with Statistical Parametric Mapping (SPM12). Structural images from all participants were skull stripped and origin corrected to improve registration precision. The initial 10 time points of each functional dataset were then discarded, with a view to allowing signal stabilization and participant adaptation to scanner noise. Slice timing correction was subsequently applied. The mean functional image served as the reference for realignment to correct interscan head motion, thereby ensuring spatial consistency across time points and reducing motion‐related artifacts. Head motion parameters were recorded for quality control, and datasets with excessive motion, defined as maximum translation exceeding 3 mm or maximum rotation exceeding 3°, were excluded from further analysis. Functional data preprocessing included a two‐step spatial registration procedure. First, each participant's T1‐weighted structural image was coregistered to the corresponding mean BOLD image. The coregistered structural image underwent segmentation to distinguish gray matter, white matter, and cerebrospinal fluid. The gray matter segment was then normalized to the MNI standard space tissue probability map, a process essential for deriving nonlinear transformation parameters. These parameters were applied to the motion‐corrected functional volumes to achieve spatial normalization. The standardized functional images were then resampled to a voxel size of 3 × 3 × 3 mm. Prior to temporal filtering, covariate regression was performed to remove signals from white matter and cerebrospinal fluid, as well as 24 head motion parameters based on the Friston‐24 model, thereby reducing non‐neuronal contributions. Subsequently, the residual time series was subjected to bandpass filtering, with the range set between 0.01 and 0.08 Hz. This was followed by spatial smoothing using a Gaussian kernel with a full width at half maximum of 4.5 mm.

### 2.4 BEN Calculation

Entropy maps of the whole brain should be generated for each participant, using the Brain Entropy Mapping Toolbox (BENtbx; Wang et al. 2014). First, the BOLD time series of each voxel were extracted from the standardized rs‐fMRI data, and the sample entropy (SampEn) method was applied to measure the complexity of the time series. To ensure calculation stability, an embedding dimension of 2 and a tolerance of 0.2 were chosen (Richman and Moorman 2000). This tolerance serves as a cutoff, representing the maximum allowable variation in the time series during computation. More information about BEN calculations can be found in the original BENtbx paper (Wang et al. 2014). Subsequently, all computed BEN values were spatially smoothed using a smoothing kernel of 4.5 mm to reduce noise and improve image quality. Finally, voxel‐wise BEN value maps were obtained for subsequent statistical analysis and regional comparisons.

### 2.5 Statistical Analysis

Statistical analysis was performed using SPSS 26.0. Qualitative data were described using frequency and percentage (*n*, %), with intergroup comparisons conducted via chi‐square test or Fisher's exact test. Quantitative data underwent Kolmogorov–Smirnov normality testing, with normally distributed data expressed as mean ± standard deviation and analyzed using independent samples *t*‐test. Non‐normally distributed data were described using median (P_25_, P_75_) and analyzed using the Mann–Whitney *U*‐test. For voxel‐level group comparisons, a two‐sample *t*‐test analysis was performed using SPM12. Given the significant group differences in age and education level observed in the demographic data (Table 1), which are known to influence BEN (Z. Wang and Alzheimer's Disease Neuroimaging Initiative 2020), we included age, sex, and years of education as nuisance covariates (regressors of no interest) in the general linear model (GLM) to control for their potential confounding effects. This ensures that the observed BEN differences between groups can be more confidently attributed to AD pathology rather than to demographic disparities.

**TABLE 1:** Demographic and clinical characteristics of the study sample.

Variable	AD group (*n* = 30)	HC group (*n* = 46)	*p* value
**Sex, male, *n* (%)**	16 (53.3%)	26 (56.5%)	0.785
**Age, median (IQR)**	63.00 (54.00, 69.25)	53.50 (50.00, 56.00)	< 0.001**
**Years of education (year)**	8.83 ± 3.63	12.89 ± 2.55	< 0.001**
**MMSE**	24.00 (21.00, 25.00)	26.00 (25.00, 28.00)	< 0.001**
**MoCA**	20.66 ± 2.69	24.78 ± 2.63	< 0.001**
**ADAS‐Cog**	27.02 ± 9.47	17.73 ± 6.05	< 0.001**
**ADL**	65.66 ± 14.27	83.45 ± 11.62	< 0.001**

Abbreviations: ADAS‐Cog, Alzheimer's Disease Assessment Scale–Cognitive Subscale; MMSE, Mini‐Mental State Examination; MoCA, Montreal Cognitive Assessment.

Data are presented as mean ± standard deviation.

A *p* value < 0.05 denotes statistical significance, and *p <* 0.001 denotes high statistical significance.Indicators (**): express *p*<0.001.

## 3 Results

### 3.1 Demographic and Clinical Characteristics

As summarized in Table 1, no statistically significant differences were observed between the AD and HC groups with respect to sex distribution (*p* > 0.05). In contrast to the HC group, the AD group displayed considerably lower MMSE, MoCA, and ADL scores, whereas ADAS‐Cog scores were markedly elevated.

### 3.2 Differences in BEN Between AD and HC Groups

To evaluate voxel‐wise differences in BEN between the AD and HC groups, two‐sample *t*‐tests were performed in SPM12. Two directional contrasts were tested separately (AD < HC and AD > HC). For each directional contrast, maps were thresholded at voxel‐wise *p* < 0.001 (uncorrected, one‐tailed; equivalent to two‐tailed *p* < 0.002 when considering both directions), with GRF/RFT cluster‐level correction at *p* < 0.05 (FWE‐corrected). Under these thresholds, the AD group showed significantly reduced BEN relative to HC (AD < HC) in three clusters located in the right superior temporal gyrus (peak MNI: 70, −24, 14), the pars opercularis of the right inferior frontal gyrus (peak: 44, 12, 6), and the junction of the left posterior parietal gyrus and superior temporal gyrus (peak: −60, −24, 18). No significant clusters were observed for the opposite contrast (AD > HC) after GRF/RFT correction.

Across all clusters, BEN values were found to be considerably diminished relative to HC values in the AD cohort. This finding indicates a reduced neural signal complexity in individuals diagnosed with AD. Error bars indicate the standard error of the mean.

**FIGURE 1:**
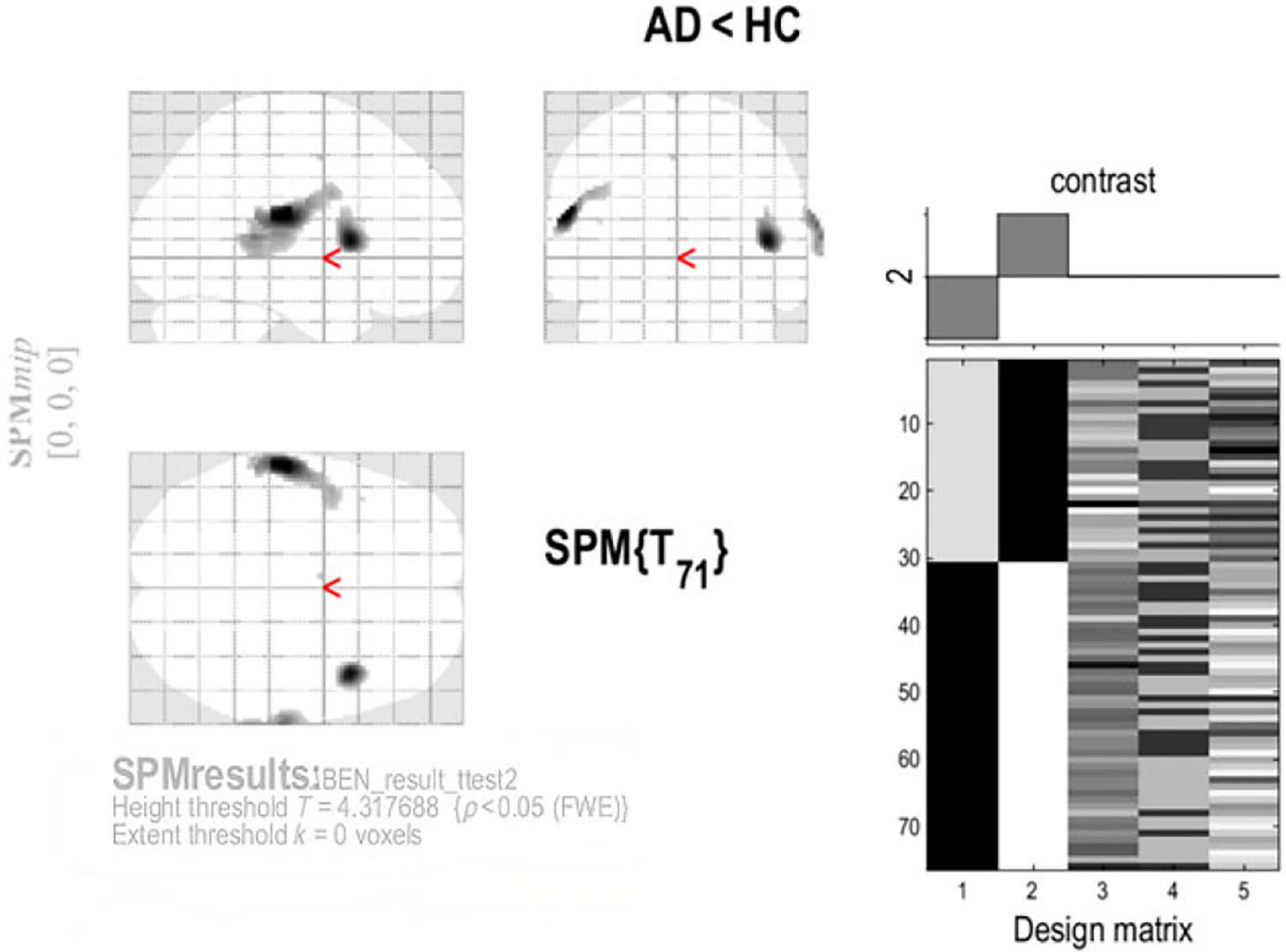
Voxel‐level comparison of BEN between AD and HC groups (FWE‐corrected). *Note*: Data were processed using SPM12, and a two‐sample *t*‐test (two‐tailed) was conducted, with a voxel‐level statistical threshold of *p* = 0.001 (one‐tailed), which corresponds to a two‐tailed threshold of *p* = 0.002. Statistical maps were corrected using GRF (FWE‐corrected, *p* < 0.05), with a smoothing kernel of 4.5 mm FWHM.

**FIGURE 2:**
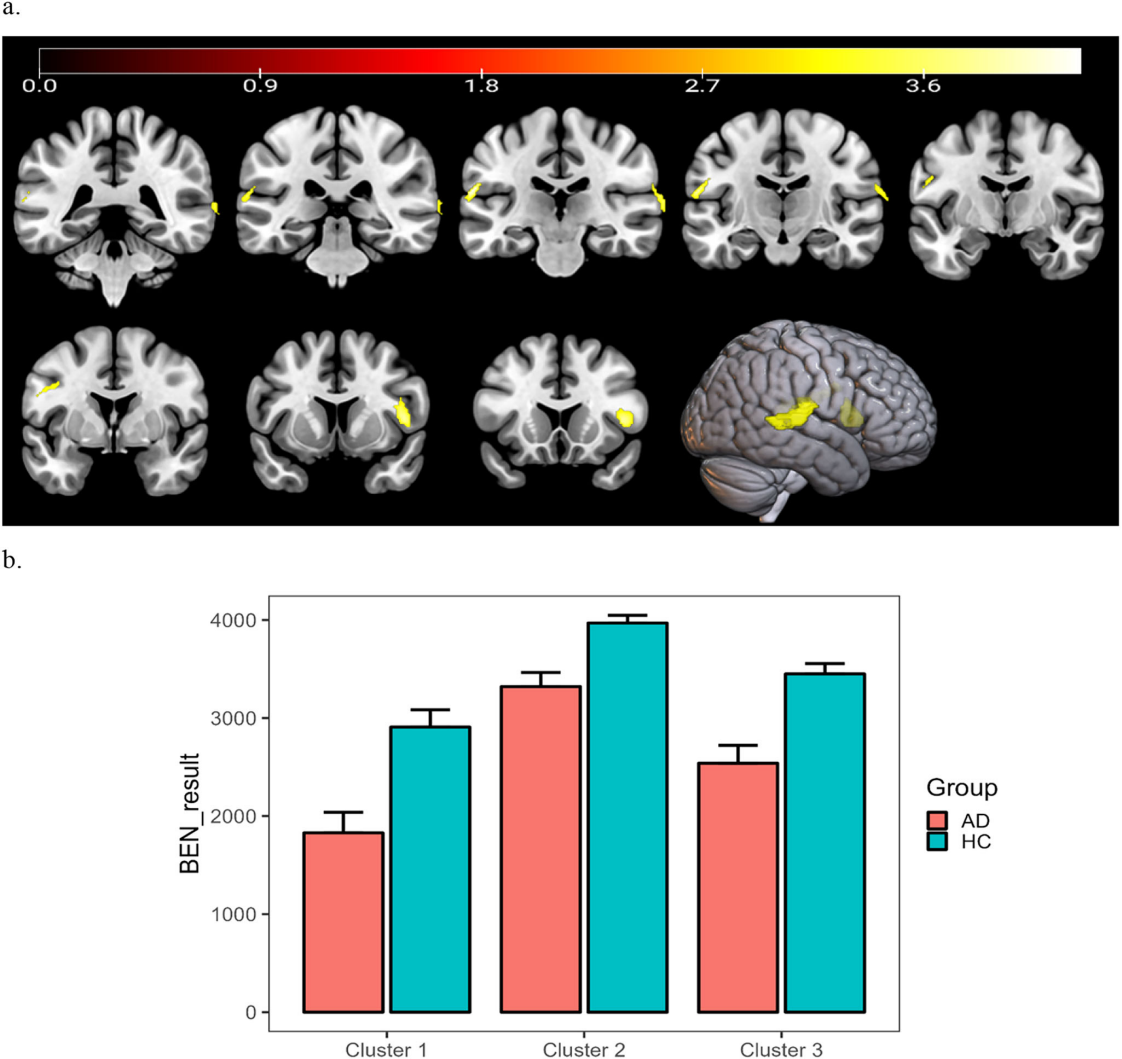
Spatial distribution of significant clusters of BEN differences between the AD group and the HC group and the mean BEN values of significant clusters. *Note*: (a) illustrates the spatial distribution of clusters exhibiting significant BEN differences between the AD and HC groups, with warm‐colored regions indicating lower BEN values in the AD group. (b) presents the mean BEN values extracted from three clusters showing significant group differences. The bar plot summarizes mean BEN values derived from the voxel‐wise analysis for cluster 1 (right superior temporal gyrus), cluster 2 (pars opercularis of right inferior frontal gyrus), and cluster 3 (conjunction area of the left posterior parietal gyrus and superior temporal gyrus). Across all clusters, BEN values were found to be considerably diminished relative to HC values in the AD cohort. This finding indicates a reduced neural signal complexity in individuals diagnosed with AD. Error bars indicate the standard error of the mean.

In comparison with the HC group, the AD group demonstrated a marked decrease in BEN across multiple brain regions (AD < HC; GRF correction; voxel‐level *p <* 0.001, cluster‐level *p <* 0.05; Figures 1 and 2). Whole‐brain voxel‐wise analysis identified three significant clusters located in the right superior temporal gyrus (peak MNI coordinates: 70, −24, 14), the pars opercularis of the right inferior frontal gyrus (peak: 44, 12, 6), and the junction of the left posterior parietal gyrus and superior temporal gyrus (peak: −60, −24, 18). The corresponding peak coordinates, *T* values, and cluster sizes are summarized in Table 2. Additionally, we explored other brain regions, including the precuneus, cuneus, and left frontotemporal pole, which showed potential correlations with cognitive performance, though they did not survive the initial group comparison threshold.

**TABLE 2:** Brain regions showing significantly decreased BEN in AD compared with HC (FWE‐corrected).

Brain region	MNI peak coordinate			*T* value	Cluster size	Cluster‐level *p* (FWE‐corrected)	Cohen's *d*
	*X*	*Y*	*Z*				
Right superior temporal gyrus	70	−24	14	3.76	202	0.003	0.87
Right inferior frontal gyrus	44	12	6	4.25	242	0.020	0.99
Left posterior parietal gyrus−superior temporal gyrus junction	−60	−24	18	4.35	272	0.045	1.01

### 3.3 Association Between BEN and Cognitive Function (Whole‐Brain Correlation Distribution and ROI Analysis)

**FIGURE 3:**
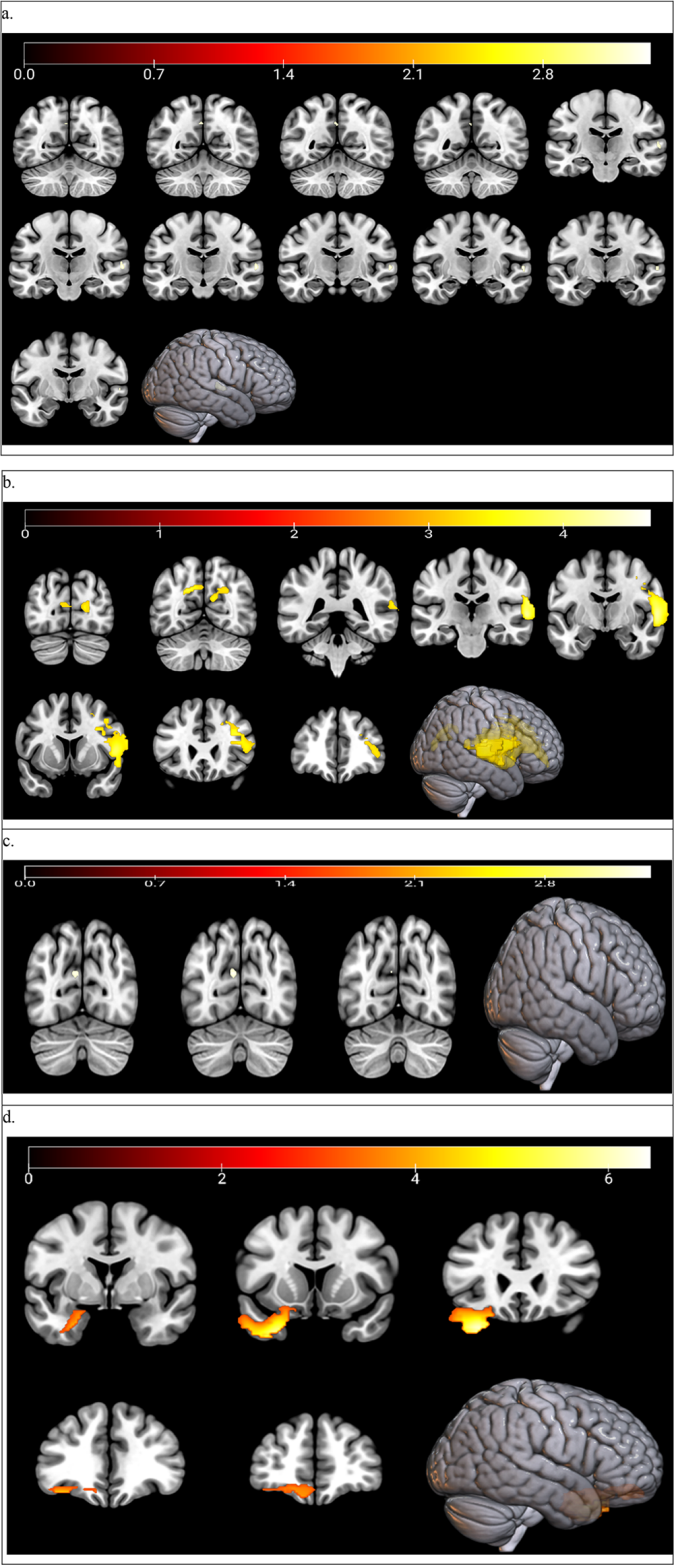
Presents voxel‐wise whole‐brain correlation maps, with each slice corresponding to a distinct anatomical level. *Note*: Subplots (a–d) display SPM‐based whole‐brain correlation maps for individual cognitive scales, in which warm colors denote positive correlations and cool colors indicate negative correlations (GRF‐corrected; voxel‐level *p <* 0.001, cluster‐level *p <* 0.05).

**FIGURE 4:**
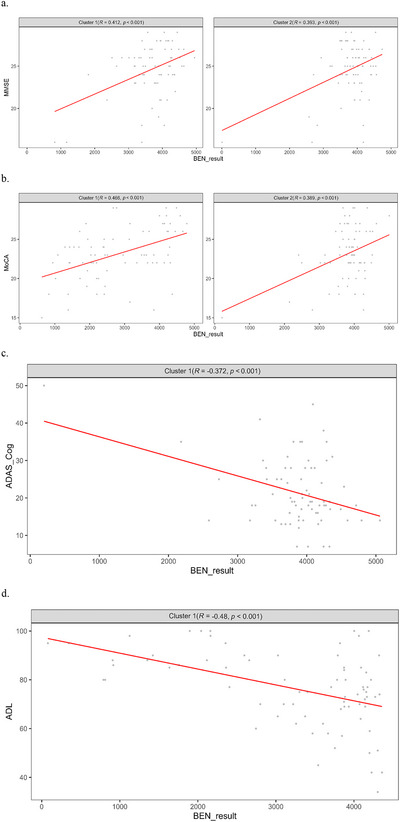
Scatter plots illustrating correlations between BEN and cognitive scale scores in representative brain regions.

Figure 3 illustrates the voxel‐level correlation patterns between BEN and MMSE, MoCA, ADAS‐Cog, and ADL scores across the entire brain. To provide a clearer depiction of these relationships in representative regions, mean BEN values were extracted from the significant clusters identified in Figure 3, and scatter plots were generated to depict associations between BEN and corresponding cognitive scale scores (Figure 4).

Figure 4a shows positive associations between BEN and MMSE in (cluster 1) the right superior temporal gyrus (partial *r* = 0.412, *p* < 0.001) and (cluster 2) the left precuneus (partial *r* = 0.393, *p* < 0.001). Figure 4b shows positive associations between BEN and MoCA in (cluster 1) the right inferior frontal gyrus–superior temporal gyrus network (Temporal_Sup_R/Frontal_Inf_Oper_R/Rolandic_Oper_R; partial *r* = 0.466, *p* < 0.001) and (cluster 2) the bilateral precuneus–cuneus region (Cuneus/Precuneus/Calcarine; partial *r* = 0.389, *p* < 0.001). Figure 4c depicts a negative correlation between BEN in the pericalcarine cortex surrounding the left calcarine sulcus (Calcarine_L; partial *r* = −0.372, *p* < 0.001) and ADAS‐Cog. Figure 4d illustrates a negative correlation between BEN in the left frontotemporal network and ADL (partial *r* = −0.48, *p* < 0.001). Partial correlations were controlled for age, sex, and years of education (Table 3).

**TABLE 3:** Partial correlation results between BEN and cognitive scales.

Brain region	Scale	MNI peak coordinate			Cluster size	Partial *r*	*p* value
		*X*	*Y*	*Z*			
Right superior temporal gyrus	MMSE	60	−16	4	43	0.412	< 0.001
Left precuneus	MMSE	−2	−56	34	13	0.393	< 0.001
Right inferior frontal gyrus/superior temporal gyrus	MoCA	60	−14	4	4757	0.466	< 0.001
Precuneus/cuneus	MoCA	−18	−62	32	855	0.389	< 0.001
Left calcarine gyrus	ADAS‐Cog	−6	−70	22	17	−0.372	< 0.001
Left frontotemporal pole network	ADL	−30	22	−38	2300	−0.48	< 0.001

For global cognitive performance, BEN in the right superior temporal gyrus and left precuneus regions exhibited significant positive correlations with MMSE scores (*r* = 0.393–0.412, *p* < 0.001), while BEN within the right inferior frontal gyrus–superior temporal gyrus network and bilateral precuneus–cuneus regions demonstrated significant positive associations with MoCA scores (*r* = 0.389–0.466, *p* < 0.001), indicating that greater complexity in auditory–language‐processing areas and DMN‐related regions corresponds to more preserved overall cognitive function. Regarding disease severity and functional capacity, BEN in the pericalcarine cortex of the left calcarine sulcus showed a significant negative correlation with ADAS‐Cog scores (*r* = −0.37, *p* < 0.001), and BEN in the left frontotemporal pole network (left superior temporal pole, left middle temporal pole, left inferior orbital frontal, left rectus, left inferior temporal gyrus) was negatively correlated with ADL scores (*r* = −0.48, *p <* 0.001), suggesting that reduced complexity within frontal and limbic system–associated networks is closely linked to cognitive decline and functional impairment.

### 3.4 Analysis of Results

A significant positive correlation with MMSE scores was observed for BEN in the right superior temporal gyrus, corresponding to the auditory language region (*r* = 0.412, *p <* 0.001), and in the precuneus, a core component of the DMN (*r* = 0.393, *p <* 0.001). BEN within the right inferior frontal gyrus–superior temporal gyrus network (*r* = 0.466, *p <* 0.001) and bilateral precuneus–cuneus regions (*r* = 0.389, *p <* 0.001) was also positively correlated with MoCA scores. In contrast, BEN in the cortex surrounding the left calcarine sulcus demonstrated a significant negative association with ADAS‐Cog scores (*r* = −0.372, *p <* 0.001), indicating that deficits in perceptual processing and visual integration are linked to overall cognitive decline. Additionally, a negative correlation was identified between BEN in the left frontotemporal network and ADL scores (*r* = −0.48, *p <* 0.001), indicating that diminished daily functional ability is closely associated with degeneration of networks supporting emotional regulation, language, and memory.

## 4 Discussion

In this study, rs‐fMRI was employed to examine alterations in BEN in patients with AD and to evaluate its associations with cognitive performance. In this study, we identified significant reductions in BEN in three key regions associated with cognitive functions in AD. These regions are the right superior temporal gyrus, right inferior frontal gyrus, and left posterior parietal gyrus, all of which play crucial roles in language processing, executive function, and memory. The reduction in BEN observed in these areas was significantly correlated with cognitive performance, as measured by the MMSE and MoCA, aligning with findings from previous studies. These results highlight the potential of BEN as a marker for cognitive decline in AD. In addition to these primary findings, we also performed exploratory analyses of other brain regions, including the precuneus, cuneus, and left frontotemporal pole. Although these regions did not show significant differences in the group comparison, they have been implicated in cognitive decline in previous research on AD. The precuneus, for instance, is a key node in the DMN and is involved in memory and spatial processing. Previous studies have linked changes in the precuneus with cognitive decline, supporting its role in AD. While these regions were not statistically significant in our analysis, their inclusion provides additional insights into the broader neural networks that may be involved in AD‐related dysfunction. It is important to note that these additional findings are exploratory and require further validation. Although they did not survive the initial statistical threshold, these regions provide valuable preliminary insights into the potential neural substrates of cognitive impairment in AD. Future studies with larger sample sizes and more rigorous statistical methods are needed to confirm these findings.

Distinct patterns of BEN alteration were observed in the brains of patients suffering from AD. Z. Wang and Alzheimer's Disease Neuroimaging Initiative (2020) reported a global reduction in BEN across the AD spectrum, characterized by an inverted U‐shaped trajectory, with significant decreases in the AD group within the DMN and regions associated with the medial temporal lobe and hippocampus. Consistently, B. Wang et al. (2017) identified marked reductions in rs‐fMRI signal complexity in the frontal, temporal, and occipital lobes of patients with AD, with significant associations with cognitive performance. In addition, EEG‐based investigations have indicated that BEN may have utility in supporting the diagnosis and monitoring of AD (Escudero et al. 2006; del Mauro et al. 2025). In intergroup analyses, a significant reduction in BEN was observed in the superior temporal gyrus of patients with AD, and BEN values in this region were positively correlated with global cognitive measures (MMSE/MoCA). The superior temporal gyrus includes the nonprimary auditory cortex and has been shown to play a vital role in speech perception through its connections with auditory and language‐processing systems, functioning as a key node in language‐processing networks (Bhaya‐Grossman and Chang 2022). This region is also involved in the interpretation of emotional and social cues, acting as an integrative hub for linguistic and affective processing (Belyk and Brown 2014; Zhang et al. 2018). Reduced temporal complexity within this area suggests diminished dynamic flexibility of language–semantic integration, which may account for impairments observed in naming and comprehension tasks. Functional alterations in the right superior temporal gyrus have been linked to Aβ and tau deposition, as accumulation of these pathological proteins may disrupt neuronal function in this region, resulting in deficits in auditory processing and emotional interpretation. This observation aligns with prior evidence implicating language network involvement in cognitive decline (Butts et al. 2022). Moreover, medial temporal lobe atrophy has been shown to correlate with progression to AD dementia, with a hazard ratio of 1.68 (95% CI, 1.20–2.35; Pyun et al. 2017; Overdorp et al. 2014).

The pars opercularis of right inferior frontal gyrus constitutes a component of the frontal lobe. Intergroup analyses demonstrated a significant reduction in BEN within this region in patients with AD. The pars opercularis is implicated in executive processes and speech motor control, serving as a central node for motor planning, task execution, and regulation of speech articulation (Jaishankar et al. 2025; Wang et al. 2022). This region also contributes to higher order cognitive functions, including attention allocation, decision‐making, and action planning, and is particularly engaged during complex task switching and adaptive decision processes. Functional alterations in the right inferior frontal gyrus in AD have been closely associated with impairments in executive function and working memory. Previous studies have reported that structural atrophy in this area parallels declines in cognitive control and motor planning capacity (Heo et al. 2025). The present findings further indicate that functional connectivity within the pars opercularis of the right inferior frontal gyrus is markedly reduced in AD and exhibits significant associations with cognitive evaluation measures, such as MoCA, thereby supporting the relevance of this region to AD‐related cognitive dysfunction.

The left posterior parietal gyrus–superior temporal gyrus constitutes a junctional region between the parietal and temporal lobes. The posterior parietal gyrus is involved in fine‐grained processing of somatosensory input, spatial perception, and multisensory integration (DiGuiseppi and Tadi 2025; Raju and Tadi 2025; Andersen 1997). The superior temporal gyrus, a component of the temporal lobe, is closely associated with auditory processing, language comprehension—given that the sensory language center is located in the posterior portion of the superior temporal gyrus of the dominant hemisphere—and memory functions. Pathology affecting the temporal lobe may result in auditory deficits, impaired language comprehension, or memory disturbances. The left posterior parietal gyrus and superior temporal gyrus jointly contribute to sensory integration as well as auditory and semantic processing. By integrating perceptual information, this region supports higher order cognitive operations, including language processing, spatial cognition, and contextual interpretation, and is particularly engaged in perception–action–language tasks, with close relevance to attentional and mnemonic functions. Existing literature indicates that the left posterior parietal gyrus and superior temporal gyrus commonly exhibit atrophy and reduced functional connectivity in patients with AD, changes that are closely associated with impairments in perceptual, linguistic, and memory‐related tasks. Degeneration within these regions has also been posited as an early indicator of cognitive decline (Putcha et al. 2018). The present results align with prior observations and further demonstrate a significant association between functional alterations in this junctional region and cognitive impairment in the AD group.

In correlation analyses between BEN and neuropsychological measures, BEN values in the superior temporal gyrus and precuneus exhibited significant positive associations with MMSE scores (*T* values of 3.42 and 3.32, respectively). Alterations in these regions were closely linked to declines in MMSE performance, particularly within the AD group, in which BEN values were markedly reduced, indicating a strong association between regional functional complexity and global cognitive abilities, including memory, attention, and language. The precuneus, located within the medial parietal lobe, is closely connected to the DMN and participates in higher order cognitive operations such as visuospatial processing, contextual construction, self‐referential cognition, and memory retrieval (Dadario and Sughrue 2023). MEG studies involving healthy individuals and patients with mild cognitive impairment have demonstrated that reduced MoCA scores are accompanied by significantly attenuated precuneus activation, suggesting a link between diminished precuneus function and cognitive decline (Yokosawa et al. 2020). Comprehensive analyses of precuneus function and brain networks have further highlighted its central role as a network hub within systems such as the DMN and CEN under both physiological and pathological conditions, with particular relevance to cognitive decline, including AD (Dadario and Sughrue 2023).

In the MoCA assessment, BEN values within the bilateral precuneus–cuneus region and the right inferior frontal gyrus–superior temporal gyrus network exhibited significant positive correlations with MoCA scores (*T* values of 4.66 and 3.83, respectively). Functional connectivity within these regions was markedly reduced in patients with AD and showed close associations with MoCA performance, particularly in domains related to language and executive function. Consistent with this observation, a prior investigation in patients with tinnitus and cognitive impairment reported a strong positive correlation between MoCA scores and connectivity strength between the precuneus and the DMN (*r* = 0.809, *p <* 0.001; Rosemann and Rauschecker 2023).

Additionally, BEN values in the cortex surrounding the left calcarine sulcus demonstrated a significant association with ADAS‐Cog scores (*T* value = 3.38, *p <* 0.05). This result indicates that reduced functional complexity in the pericalcarine cortex is closely linked to cognitive decline in AD, particularly in terms of memory, language and function impairments. The cortex adjacent to the calcarine sulcus, as a component of the occipital visual cortex, can be affected during early stages of AD (Parra et al. 2024; Yang et al. 2019). Previous studies have shown that individuals with subjective cognitive decline exhibit reductions in gray matter volume within the occipital lobe, including the pericalcarine cortex, reflecting early atrophic changes (Riverol et al. 2024). Although early AD is characterized by abnormal connectivity within the visual cortex, Bick et al. (2023) demonstrated that distal functional connectivity disruptions in this region were associated with cognitive decline, further supporting the relationship between visual cortical areas, including the calcarine sulcus, and cognitive function.

Significant associations were observed between BEN values in the right inferior frontal gyrus, frontal pole, and left frontotemporal pole network as well as ADL scores (*T* = 6.44), indicating that reduced functional complexity within these regions is closely linked to daily functional capacity in patients with AD. Previous investigations have demonstrated that declines in ADL accompany the transition from MCI to AD dementia and are associated with reduced metabolic activity in the frontal and parietal lobes, along with cortical thinning of the inferior temporal region (Halawa et al. 2019). F. Zhang et al. (2024) further reported that structural integrity and functional connectivity of white matter within the inferior frontal gyrus were related to multiple cognitive domains, including attention, inhibitory control, and executive function. As an integrative network supporting semantic processing, emotional regulation, and memory, reduced functional complexity of the frontotemporal pole network may contribute to impairments in executive control, language comprehension, and affective modulation, thereby influencing ADL (Chauveau et al. 2025). Pathological deposition of Aβ and tau is likely to disrupt neural activity within the right inferior frontal gyrus, frontal pole, and left frontotemporal pole network, promoting network degeneration and subsequent declines in cognitive and functional abilities in AD. Reduced BEN may represent an early indicator of diminished information‐processing capacity in these regions, with such functional alterations exerting direct effects on patient autonomy and quality of life.

From a neurobiological perspective, reductions in BEN reflect attenuation of the complexity of local neuronal activity patterns, which may be closely linked to the core pathological processes of AD. The accumulation of Aβ and the hyperphosphorylation of tau protein hyperphosphorylation contribute to synaptic dysfunction and neuronal loss, thereby diminishing interregional functional coupling and network adaptability. In a healthy brain, neural networks typically exhibit high dynamic complexity, enabling flexible responses to external stimuli; in contrast, AD‐related networks tend to adopt more synchronized and rigid configurations, with fewer effective information transmission pathways, resulting in marked reductions in BEN. Notably, the present results indicate that BEN decreases are not confined to the DMN but also extend to the frontoparietal executive network and limbic system, suggesting that AD pathology affects a broader array of functional networks and contributes to deficits across multiple cognitive domains.

It is important to note that these additional findings are exploratory and require further validation in future studies with larger sample sizes and more stringent statistical controls. Despite their lack of statistical significance in the current analysis, these regions provide valuable preliminary insights into the potential neural substrates of cognitive impairment in AD. Future work should focus on replicating these findings and determining whether these regions exhibit functional changes in larger cohorts of AD patients.

The present findings provide meaningful implications for clinical application. First, BEN, as a functional imaging metric derived from rs‐fMRI, offers several advantages, including noninvasiveness, task‐free acquisition, and quantitative assessment, enabling objective characterization of alterations in brain functional complexity. In contrast to conventional functional connectivity analyses, BEN emphasizes the complexity and nonlinear properties of local neural signals, allowing detection of subtle early changes associated with network‐level functional decline. Second, the observed associations between BEN and cognitive scale scores indicate that BEN may serve as an objective imaging marker of cognitive decline, offering complementary diagnostic information in clinical settings. With the incorporation of longitudinal follow‐up data, BEN may further evolve into a reliable indicator for monitoring disease progression and assessing therapeutic responses in AD. Moreover, integration of BEN with other imaging methods, such as structural MRI, DTI, and PET, as well as blood or cerebrospinal fluid biomarkers, may enhance the comprehensiveness of AD evaluation.

In summary, the results demonstrate that patients with AD exhibit widespread reductions in resting‐state BEN, primarily affecting key regions including the DMN, hippocampus, and frontal and parietal cortices, and that BEN levels are closely associated with cognitive performance. These observations indicate that BEN sensitively captures declines in neural network complexity and their cognitive consequences, supporting its potential clinical utility. Nonetheless, given the limited sample size and cross‐sectional design, further validation through large‐scale longitudinal studies is required. Although BEN shows promise for early diagnosis and disease monitoring, its clinical accuracy and reliability warrant additional evaluation in combination with other neuroimaging measures and biological markers.

### 4.1 Limitations

However, it is imperative to acknowledge the existence of several limitations. First, the sample size is relatively small, and it is a single‐center study. There are imbalances in baseline age and education level between the two groups. Although these factors were included as covariates in the statistical model, they may still affect the statistical power and the generalizability of the results. Future studies should conduct large‐scale, multicenter cohort research to validate these findings. Second, the cross‐sectional nature of the study precludes the drawing of inferences regarding causal relationships between BEN alterations and cognitive decline. Longitudinal follow‐up is required to further evaluate the predictive value of BEN for cognitive decline. Third, detailed stratified analyses across distinct cognitive domains were not performed, despite the fact that AD typically involves impairments in multiple domains, including memory, executive function, language, and visuospatial abilities. Subsequent studies could address this issue by examining domain‐specific associations between BEN and cognition using more comprehensive neuropsychological batteries. Fourth, although rigorous preprocessing and quality control procedures were applied, rs‐fMRI remains sensitive to head motion and physiological noise, and residual artifacts cannot be fully excluded; further refinement of acquisition and preprocessing strategies is therefore warranted. Finally, direct comparisons between BEN and established pathological markers of AD were not undertaken, resulting in limited multimodal validation. Future work integrating PET, cerebrospinal fluid, and related biomarkers may strengthen the biological interpretability of these findings.

## Author Contributions

**Xuke Zhang, Meihai Wen**: writing – original draft, resources, project administration, methodology, investigation, formal analysis, data curation. **Simin Zhang, Chen Rao**: resources, methodology, investigation. **Zhiwen Zha, Tong Gu, Chen He**: investigation, formal analysis. **Yuanyuan Song, Shuang Niu**: investigation, data curation. **Lei Zhu**: methodology, investigation, funding acquisition, formal analysis. **Chuanqing Yu**: writing – review and editing, methodology.

## Funding

This research was supported by the Yantu Neuroscience Special Research Fund Project (Z‐2017‐24‐2509), Regional Stroke Risk Factor Early Identification and Prediction System Construction (WKZX2023CZ0115), Anhui Province Clinical Medical Research and Transformation Special Project （202304295107020051）, Hefei Comprehensive National Science Center Institute of Health Research Joint Research Center for Occupational Medicine and Health Project (OMH‐2024‐031), the Open Fund of Anhui International Joint Research Center for Nano Carbon‐based Materials and Environmental Health (Grant No. NCMEH2024Y06), Anhui University of Science and Technology First Affiliated Hospital Sanxin Project (2024‐LC‐SX059), and the Graduate Student Research Innovation Program of Bengbu Medical University (Byycxz24045).

## Ethics Statement

All human participant procedures involved in this study were conducted in accordance with the Declaration of Helsinki (2013 revision). The study protocol was approved by the Ethics Committee of the First Hospital of Anhui University of Science and Technology (Approval No: 2024‐LC‐SX059‐001).

## Consent

All the authors approved the submitted version, and all patients provided informed consent.

## Conflicts of Interest

The authors declare no conflicts of interest.

## Data Availability

The datasets generated during and/or analyzed during the current study are available from the corresponding author upon reasonable request.
